# Synergistic effect of oxaliplatin and nanocurcumin in dendrosomal carrier to inhibits ovarian cancer cells invasion and metastasis through the long non-coding RNA MEG3

**DOI:** 10.1016/j.crphar.2025.100243

**Published:** 2025-12-02

**Authors:** Elahe Seyed Hosseini, Marziyeh Alizadeh Zarei, Zahra Shabani, Hamed Haddad Kashani, Hossein Nikzad

**Affiliations:** a Anatomical Sciences Research Center, Institute for Basic Sciences, Kashan University of Medical Sciences, Kashan, Iran; b Department of Pharmacy, School of Pharmacy, Hamadan University of Medical Sciences, Hamadan, Iran

**Keywords:** Dendrosomal nanocurcumin, LncRNA, MEG3 knockdown, MMP-2, MMP-9

## Abstract

**Background:**

Ovarian cancer (OC) remains one of the most lethal gynecological malignancies worldwide. The long non-coding RNA MEG3 (Maternally Expressed Gene 3), located on chromosome 14q32.3, has been identified as a tumor suppressor in various cancers. This study investigated the impact of siRNA-mediated MEG3 silencing in the context of dendrosomal nanocurcumin (DNC) and Oxaliplatin (OXA) treatments on ovarian cancer cell lines, focusing on the expression of genes associated with apoptosis and metastasis, including Bcl-2, BAX, MMP-2, and MMP-9.

**Methods:**

Cell viability was assessed using the MTT assay; apoptosis and cell cycle progression were evaluated via flow cytometry and Annexin V-FLUOS staining. Cell migration and invasion were examined using the transwell assay, and gene expression levels were quantified by real-time PCR.

**Results:**

MEG3 expression significantly increased in both cell lines upon DNC and OXA treatment in a time-dependent manner, with the most pronounced effect in OVCAR3 cells (P < 0.01–0.001). MEG3 silencing significantly attenuated the anticancer efficacy of both agents. Notably, MMP-2 expression increased in DNC (P < 0.01) and combination therapy (P < 0.001), while MMP-9 was upregulated following OXA treatment (P < 0.01) after MEG3 knockdown.

**Conclusion:**

These results suggest that MEG3 knockdown impairs the anti-metastatic properties of DNC and OXA by modulating MMP-2 and MMP-9 expression. The combination of DNC and OXA holds promise as an effective therapeutic strategy in OC, and MEG3 may serve as a potential biomarker and therapeutic target, particularly in drug-resistant ovarian cancer.

## Highlights

• **DNC and OXA Synergy:** Combined DNC and oxaliplatin treatment strongly reduced ovarian cancer cell migration and invasion.
• **MEG3 Modulates Drug Response:** MEG3 silencing weakened the anticancer effects of DNC and OXA, highlighting its role in drug sensitivity.
• **MMP-2 and MMP-9 Regulation:** Knockdown of MEG3 increased MMP-2 and MMP-9 expression, promoting cancer cell invasion.
• **Enhanced Delivery via Nanocarriers:** Dendrosomal nanocurcumin improved curcumin's bioavailability and anticancer activity.
• **MEG3 as a Biomarker:** MEG3 may serve as a marker for treatment response and a target to overcome drug resistance.
• **Path to Precision Therapy:** Combining lncRNA targeting with nanomedicine offers a promising strategy for treating drug-resistant ovarian cancer.

## Graphical abstract

**Figure:**
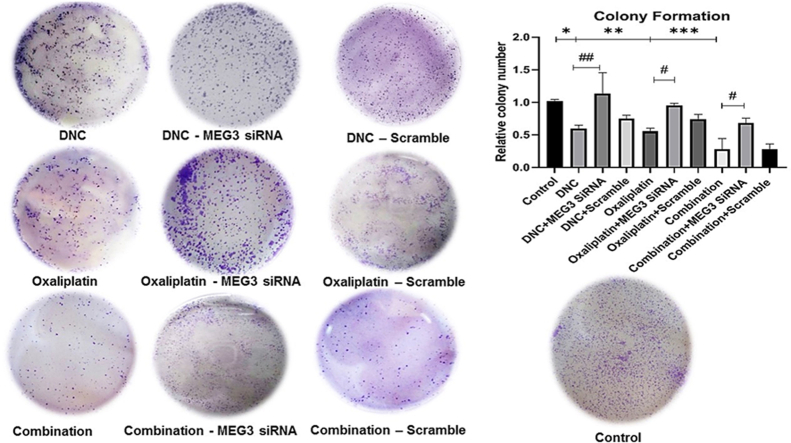

## 1 Introduction

Ovarian cancer (OC) is one of the most common cancers and the fifth leading cause of cancer-related mortality among women worldwide(Ferlay et al., 2010; Miller et al., 2016). Chemoresistance remains a major clinical challenge in the treatment of patients with ovarian cancer. Efforts to overcome this issue have recently focused on the use of novel chemopreventive agents with higher efficacy and fewer side effects in cancer therapy. Oxaliplatin (OXA), a third-generation platinum-based chemotherapeutic agent, is used clinically either alone or in combination with other anticancer drugs to treat ovarian cancer (Machover et al., 1996). More recently, to enhance the efficacy of OXA, it has been combined with natural phytochemicals such as curcumin (Cur) (He et al., 2017a; Seyed Hosseini et al., 2023). Curcumin is a natural polyphenolic compound extracted from the rhizome turmeric with potent anti-tumor effect (Shishodia et al., 2007; Gupta et al., 2013, 2022; Zarei et al., 2025). Dendrosomal curcumin (DNC) has been recently used as a novel nanoparticle formulation of curcumin, due to therapeutic limitation of curcumin such as water insolubility, low adsorption, and rapid metabolism (Zarei et al., 2025; Babaei et al., 2012). Dendrosome is an efficient neutral nano-carier synthesized firstly by Babaei et al. in laboratory at 2012 for safely delivering genes into different cell lines (Babaei et al., 2012; Sarbolouki et al., 2000). A growing number of evidences demonstrated anticancer effect of DNC and OXA combination treatments on multiple biological processes of tumor proliferation, apoptosis and cell cycle arrest in ovarian cancer (Sarbolouki et al., 2000). Recent studies have also revealed the crucial role of specific long non-coding RNAs (lncRNAs) in affecting sensitivity to the therapeutic drugs or even drug-resistant in ovarian cancer cells. Long non-coding RNAs (lncRNAs) with longer than 200 nucleotides have be found to be associated with the development of many types of cancers (Spizzo et al., 2012; Hosseini et al., 2017, 2019). Among them, maternally expressed gene 3 (MEG3) is known for its contribution in response to chemotherapeutic agents such as curcumin and OXA (Ghafouri-Fard and Taheri, 2019). MEG3 is a1.6 kb long non-coding RNA (lncRNA) located on chromosome 14q32.3 and consists of ten exons (Zhang et al., 2010). Previous studies have illustrated that MEG3 may affect proliferation and apoptosis of tumor cells (Hosseini et al., 2021; Qin et al., 2013; Wang et al., 2012; Ying et al., 2013; Zamani et al., 2014). However, little is known about the role of MEG3 in the development of chemo resistant in ovarian cancer. Based on results of our previous study, the combination of DNC and OXA had a synergistic inhibitory effect on the expression levels of a panel of long non-coding RNAs and affecting cell proliferation and apoptosis in OVCAR3 cell lines (Hosseini et al., 2019). Earlier studies in our research group found the anticancer properties of DNC are mediated by regulating the expression of lncRNA MEG3 and apoptotic-associated markers of Bcl-2 and BAX in ovarian cancer (Zamani et al., 2015). Several studies have also reported that curcumin exert its anti-cancer activity in cancer cells via interacting with a variety of cancer –associated molecular targets such as BAX, BCL-2, MMPs (MMP-9, MMP-2), cyclin D1, TP53 via modulating signaling pathways involved in cancer development (Choudhuri et al., 2005; Kunnumakkara et al., 2008). Matrix metalloproteinase (MMPs) such as MMP-2 and MMP-9 facilitate tumor progression and EMT-associated metastasis directly by degrading the basement membrane components, allowing cancer cells to invade into the surrounding (Li et al., 2015; Fanelli et al., 2012; Sato et al., 1994; Radisky and Radisky, 2010). Better understanding the mechanisms of chemotherapeutic drug resistance and chemo sensitivity to DNC and OXA in ovarian cancer cell lines, facilitate the development of more effective anti-tumor drugs, and thereby improve the survival rate of the patients. Therefore, the aim of this study is to further explore of mechanisms underlying the role of MEG3 expression levels on anti-cancer effects of DNC, OXA and combination treatment, via meditating MMP-2 and MMP-9 involved in tumor cell invasion and metastasis in OVCAR3 and SKOV3 ovarian cancer cell lines.

## 2 Methods and materials

### 2.1 Cell lines and culture conditions

OVCAR3 and SKOV3 ovarian cancer cell lines used in this study were purchased from Pasteur Institute of Iran (Tehran, Iran). The cells cultured in RPMI 1640 medium (Invitrogen, USA) supplemented with 10 % fetal bovine serum (FBS) (Invitrogen, USA) and 100 units/ml penicillin–streptomycin (Gibco, Scotland) in 5 % CO2 at 37*°C* in a humidified incubator.

### 2.2 Agents

OXA and Cur powders was purchased from Merck (Darmstadt, Germany) with purity of 95 %. OXA was initially dissolved in dimethyl formamide (DMF) followed by dilution with milli Q (mQ) water (at a ratio 1:5) to produce a 1 mM stock solution.

### 2.3 Dendrosome (polymeric micelle OA400 carrier) preparation

Dendrosomal nanoparticles (Den 400) and DNC (a combination of curcumin powder and liquid dendrosome), were prepared based on previous protocol (Babaei et al., 2012; Hosseini et al., 2019; Sadeghizadeh et al., 2008). Briefly, the OA400 carrier (Dendrosome nanoparticle specified Den 400) was synthesized by esterification of oleoyl chloride (0.01 mol) and polyethylene glycol 400 (0.01 mol) in the presence of triethyl amine (0.012 mol) and chloroform as the solvent at 25 ^°^C for 4 h. After filtration of trimethylamine hydrochloride salt from organic phase, chloroform was eliminated from OA400 through evaporation in a vacuum oven at 40 ^°^C for 4 h. For DNC preparation, different weight/weight ratios of dendrosome/curcumin (about 50:1 to 10:1) were investigated by spectrophotometry (Infnite®200 PRO, Tecan, Mannedorf, Switzerland) in order to select an appropriate ratio of 1:25 as the optimum ratio. The curcumin was dissolved in dendrosome nanoparticles using protocols Maling Gou et al. (2011) Prepared DNC at a concentration of 2700 mM was kept at 4 °C and kept away from light until use. For in vitro experiments, DNC was diluted in culture medium before use in any assay. Fourier transform infrared (FTIR) Spectrum analysis was performed to confirm the dendrosomal chemical structure. Dynamic light scattering (DLS) analysis confirmed the mean diameter of ≤200 nm for DNC and transmission electron microscopy (TEM) exhibited DNC nanoparticles were sphere shaped. Also, the efficient encapsulation of curcumin in the dendrosomal nano-carrier was very high (87 %). Additionally, acceptable value of the superficial charge (ζ-potential) was calculated around −7 mV which does not show high stability.

### 2.4 Small interfering RNA to knockdown long non-coding RNA MEG3

For the MEG3 knockdown experiments, the ovarian cancer cell lines OVCAR3 and SKOV3 were seeded into six-well plates in RPMI-1640 medium for 24h. The BLOCK-iT RNA interference (RNAi) designer was used for designing of four different Small Interfering RNAs (siRNAs) that targeted MEG3 RNA with the following sequences.

1-5-GCUGUCCCUCUUACCUAAA-3
2- 5-GCAUUAAGCCCUGACCUUU-3
3- 5-GGAAGGAUCCCUUUGGGAA -3
4- 5- GCUAGCAAACUGGAGUGUU -3

and a scrambled siRNA control were purchased from Life Technologies. The MEG3 siRNAs stored at −20 °C after dissolving in RNase free-water, the cells were transfected with these siRNAs (oligonucleotides encoding 19-mer hairpin sequences) with lipofectamine 3000 (Invitrogen) specifically targeting MEG3, according to the manufacturer's instructions. Transfection efficacy was checked with scramble for 24, 48 and 72h with GFP florescent. 48h after transfection was a best time of transfection efficacy. Briefly, cells were plated in 6-well culture dishes and allowed to attachment. The experimental conditions included DNC, DNC + MEG3 siRNA, OXA + MEG3 siRNA, co-treatment with DNC and OXA + MEG3 siRNA, and untreated cells as controls. Cells were treated with DNC, OXA, or their combination for 48 h prior to MEG3 siRNA transfection. The efficiency of siRNA-mediated knockdown was confirmed by real-time PCR analysis.

### 2.5 MEG3 expression analysis by real-time PCR

Total RNA was extracted from OVCAR3 and SKOV3 cells using TRIzol® reagent ((Invitrogen Life Technologies) followed by DNase I digestion (Thermo Fisher Scientific, Waltham, MA, USA). The quantity and quality of the isolated RNA were determined by Nanodrop ND-1000 (Nanodrop Technologies, Wilmington, Delaware, USA) and agarose gel electrophoresis (1 % agarose; Gibco/BRL), respectively. 500 ng of extractive RNA was used to synthesize the cDNA by PrimeScript™ RT reagent kit (Takara Bio Inc., Shiga, Japan). Real-time PCR was performed by IQ5 (Bio-Rad, Germany) using 2 μl of the synthetized cDNA and SYBR green master mix (Biofact, Corea) in a total reaction volume of 10 μl. PCR was performed in triplicate. GAPDH, as the internal control, was used to normalize the gene expressions. The sequences of specific primers were illustrated in Table 1. The relative expressions were calculated according to 2−ΔΔCt.

**Table 1:** List of specific primers used in real-time polymerase chain reaction assay.

GENE	DESIGNED OLIGONUCLEOTIDE	AMPLICON LENGTH (BP)
MEG3	TCCGTCCACCTCCTTGTCT	233
	TAGGGCATTGGTTTAAGTCTTTAG	
BCL2	GGGATGCGGGAGATGTGG	236
	GTAGCGGCGGGAGAAGTC	
BAX	AAGAAGCTGAGCGAGTGTCT	236
	GTTCTGATCAGTTCCGGCAC	
MMP2	TTGATGGCATCGCTCAGATC	175
	TTGTCACGTGGCGTCACAGT	
MMP9	GACGCAGACATCGTCATCCA	190
	CACAACTCGTCATCGTCGAAA	
GAPDH	GAGTCAACGGATTTGGTCGT	237
	TTGATTTTGGAGGGATCTCG	

### 2.6 Colony formation assay

Colony formation assay was conducted to evaluate the role of MEG3 in the cell proliferative potential of OVCAR3 and SKOV3 cells. For this assay, the cells were seeded into 6-well plates at 500 cells/well and transfected with MEG3 siRNA in optimum and lipofectimin. Then, the cells were treated with DNC, OXA or combination of them at concentrations lower than its IC50 values for 48h then cells were transfected with MEG3 siRNA for 48h (Qin et al., 2013). After that, the cells were cultured at 37 °C and 5 % CO2 in incubator. After 10 days, the dishes were washed twice in PBS, fixed by methanol and stained with 0.1 % crystal violet and then air dried. The total number of colonies was counted in a microscope. The percentage of colonies was defined with the number of colonies formed in treated plate divided by number of colonies formed in control groups with no treatment.

### 2.7 Cell apoptosis assay

Cell apoptosis assay was performed to detect the apoptosis rate by using Annexin V-FITC/PI apoptosis detection kit (Roche Applied Science, Penzberg, and Germany.) was used to detect the apoptosis rate according to the manufacturer's instruction. Briefly, the OVCAR3 cells were seeded in 6-well plates overnight, then treated similar to above. After washing twice with PBS, treated cells were stained with Annexin V-FITC/PI (Roche Applied Science, Penzberg, Germany cat number: 858777001), finally incubated for 10–15 min at 15–20 °C and analyzed by FACSCalibur flow cytometer immediately.

### 2.8 Transwell cell invasion assays

Cell invasion of ovarian cancer cells was determined by Transwell Filter with 8 μm pore size coated with Matrigel (BD Biosciences, San Jose, CA, USA). Briefly, poly vinyl pyrolidone-free polycarbonate filters (Millipore) (8μ M pore size) were coated with matrigel (15 μg/filter). 48h after treatments and MEG3 siRNA transfection, OVCAR3 cells in 200 μl of serum-free DMEM were seeded into the upper chamber, while medium supplemented with 10 % FBS was added to the lower chamber which acts as a chemo attractant. After 48 h incubation, the non-invading cells remaining in the upper chamber were removed with a cotton-tipped swab. The invaded cells on the lower surface of the membrane were fixed and stained by methanol and 1 % crystal violet, respectively. Then, data were recorded through direct observation and the cells were photographed under a microscope. Finally, the number of invasive cells was counted using a light microscope in at least 10 random fields for each well.

### 2.9 Transwell migration assays

The migration assay was identical to above invasion assay except the inserts were not coated with Matrigel. A 24-well transwell chamber with 8.0-μm pore size membrane was used according to the manual Instructions. In brief, 48h after treatments and MEG3 siRNA transfection, the cells were suspended in culture media and were seeded into the upper chamber of the transwell plates. After 24h incubation in at 37 °C, the non-migrated cells on the upper surface of the membrane were removed with a cotton swab. The migrated cells to the lower surface of the membrane were then fixed and stained with methanol and 1 % crystal violet, respectively. The cells were counted in 10 randomly separate fields per membrane using a microscope.

### 2.10 Cell cycle assay

Cell cycle was detected by flow cytometry assay. For the cell cycle assessment, 1.5 × 10^5^ cells/well of OVCAR3 were seeded 6-well plates overnight at 37 °C and 5 % CO2. After overnight of incubation, the cells were treated with DNC, OXA and combination of them at the proper concentration. All experiments were repeated three times. 48h after treatments, cells were transfected with MEG3 siRNA. Finally, after 48h, the cells were washed twice with PBS, harvested and then trypsinesed and fixed in 70 % cold ethanol and incubated at 20 °C. After washing the cells twice with PBS, they were suspended in a solution containing 10 mg/ml propidium iodide (Molecular Probes, Invitrogen, UK) and 0.2 mg/ml RNAase-DNAase free (Sigma) and incubated for 30 min at 37 °C in the dark. The percentage of cells in the G0/G1, S, and G2/M phases were determined by a flow jo software for Windows 64-bit (Beckman Coulter, USA). Gating strategy was done to exclude cell doublets, clumps and debris.

### 2.11 Statistical analysis

All experiments were repeated at least three times. Statistical analysis was performed by SPSS 16.0 software (SPSS Inc., USA). One-way ANOVA and Tukey's test were performed for multi-group comparison. All the data are presented as mean ± SD and value of P < 0.05 was considered statistically significant.

## 3 Results

### 3.1 DNC nanoparticles and oxaliplatin increased MEG3 expression in ovarian cancer cells

**Fig. 1:**
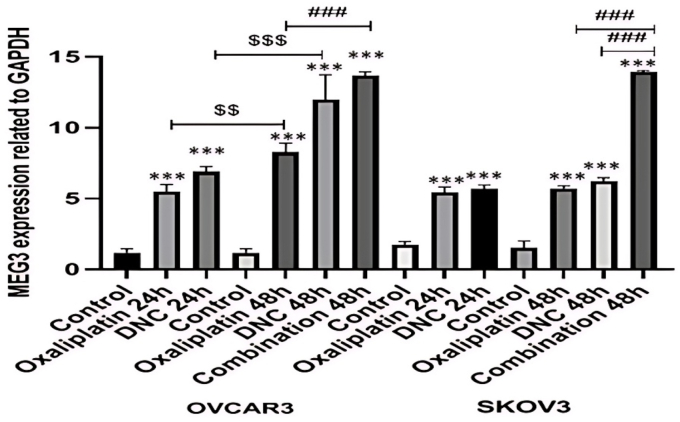
**effect of dendrosomal nanocurcumin (DNC), oxaliplatin (OXA), and their combination on MEG3 gene expression after 24h and 48h treatments in ovarian cancer cell lines (OVCAR3 and SKOV3).** Notes: Gene expression levels were measured by real-time PCR and normalized to GAPDH. Data are presented as mean ± standard error of the mean (SEM) from three independent experiments (n = 3) for each treatment group. ∗∗∗P ≤ 0.001 compared to control group, ###P ≤ 0.001 compared to combination group, $$P ≤ 0.01, $$$P ≤ 0.001 between 24h and 48h time points.

In this study, we observed MEG3 expression was increased in OVCAR3 and SKOV3 cell after all 3 treatments with DNC, OXA and combination of them at 24–48h. Also, this MEG3 over-expression was in a time-dependent manner and higher after 48 h treatment with OXA (P < 0.01) in OVCAR3 cell lines. Notably, the higher MEG3 expression in OVCAR3 cell line was significant only in combination treatment compared to the DNC treatment alone (P < 0.001) (Fig. 1). We also assessed the role of siRNA-mediated knockdown of MEG3 on induced apoptosis, anti-metastatic, invasion and cell cycle arrest effects of DNC treatment alone, also in combination with OXA in OVCAR3 cell lines.

### 3.2 MEG3‏ ‏down regulation arrested anti cell proliferative effect of DNC nanoparticles and oxaliplatin in ovarian cancer cells

**Fig. 2:**
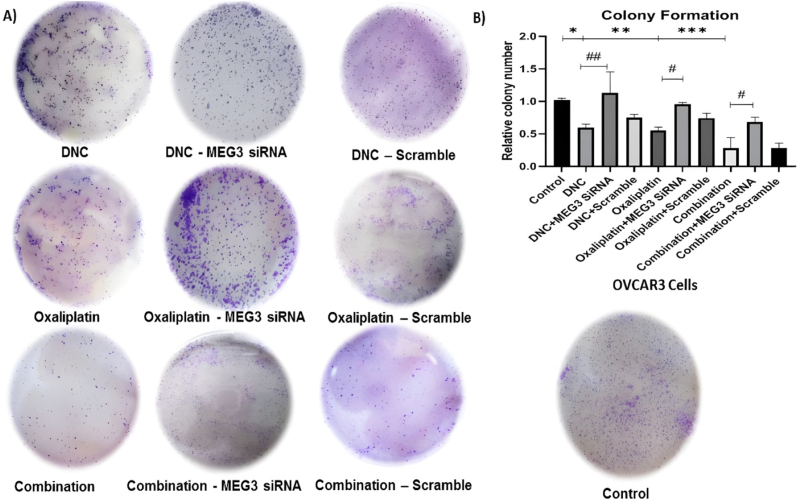
**Effect of MEG3 silencing on the ability of DNC, oxaliplatin (OXA), and combination therapy to inhibit colony formation in OVCAR3 ovarian cancer cells**. **(A)** Representative images of colonies after the indicated treatments. **(B)** Quantification of relative colony numbers from three independent experiments (mean ± SEM in three independent experiments (n = 3). ∗P ≤ 0.05, ∗∗P ≤ 0.01, ∗∗∗P ≤ 0.001 vs. untreated control group, #P ≤ 0.05, ##P ≤ 0.01 vs. corresponding treatment group with MEG3 siRNA.

Based on previous study, we had shown a significant decrease in cell proliferation and colony formation in ovarian cancer cell lines after treatment with DNC, OXA and combination of them compared to control untreated cells. This significantly reduction in cell proliferation was higher in combination therapy (P < 0.001) than DNC (P < 0.05) or OXA (P < 0.01) treatment alone. Our data demonstrated that MEG3 knockdown could significantly decline anti proliferative effect of all treatments. After MEG3 siRNA the colony formation in ovarian cancer cell line was increased from 0.55 to 1.1 in DNC (p < 0.01), 0.5 to 0.95 in OXA (p < 0.05) and 0.3 to 0.6 in combination treatment (p < 0.05). The above results suggested that inhibitory effect of all treatments on colony formation in ovarian cancer cell lines was influenced by MEG3 downregulation (Fig. 2).

### 3.3 MEG3 down regulation inhibited apoptotic activating effect of DNA and oxaliplatin treatment in ovarian cancer cells

**Fig. 3:**
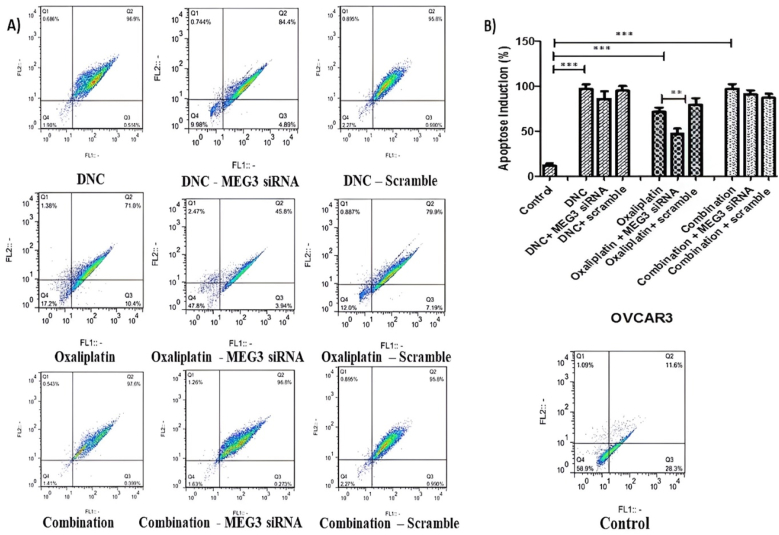
**Effect of MEG3 silencing on apoptosis induction in OVCAR3 ovarian cancer cells**. **(A)** Flow cytometry scatter plots showing the percentage of apoptotic cells (Q2 and Q3 quadrants) after 48 h of treatment with DNC, OXA, or their combination. The effect of siRNA-mediated MEG3 knockdown was compared with each respective treatment and the scrambled siRNA control. **(B)** Quantitative analysis of apoptosis induction. The bar graph presents the mean percentage of apoptotic cells ± standard deviation from three independent experiments (n = 3). Treatments were compared with the untreated control (∗∗P ≤ 0.01, ∗∗∗P ≤ 0.001). Statistical significance between each treatment group and its corresponding MEG3 siRNA-knockdown group is also indicated (###P ≤ 0.001).

As our previous result (Qin et al., 2013; Wang et al., 2012), we observed a significantly induced apoptosis in OVCAR3 cell lines treated with DNC, OXA and a combination of them compared with untreated control group (P < 0.001). We further explored that OXA treatment combined with MEG3 downregulation could significantly decrease the percentage of the apoptotic cell from 75 % to 50 % compared with OXA treatment alone (P < 0.01). However, this synergistically effect was not observed in the other two treatments. In other words, OXA-induced apoptosis is partly influenced by the MEG3 downregulation in ovarian cancer (Fig. 3).

### 3.4 MEG3 down regulation inhibited anti-migrate effect of DNC nanoparticles and oxaliplatin in ovarian cancer cell

**Fig. 4:**
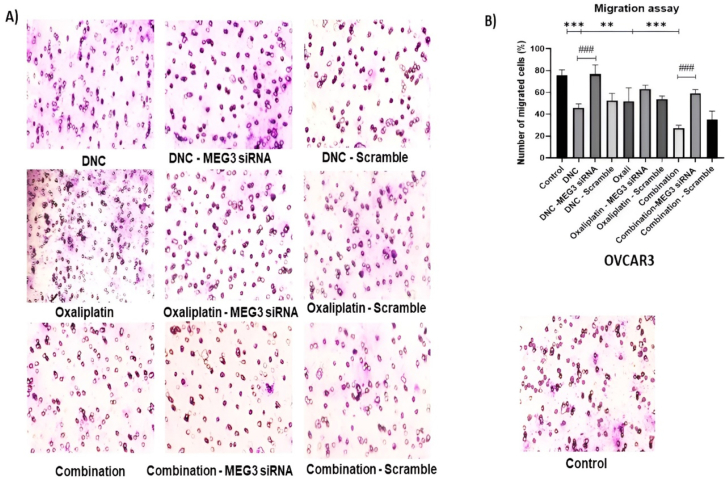
**Impact of MEG3 silencing on the migration of OVCAR3 ovarian cancer cells.** (A) Representative images of migrated cells in the Transwell assay for each treatment group. (B) Quantitative analysis of cell migration. The graph shows the mean percentage of migrated cells ±standard deviation. The effects of DNC, OXA, and their combination were evaluated with and without siRNA-mediated MEG3 knockdown. Statistical significance was determined by comparing treated groups to the untreated control∗∗P ≤ 0.01, ∗∗∗P ≤ 0.001 Significance between a treatment group and its corresponding MEG3 siRNA-knockdown group is also shown., ###P ≤ 0.001 Note: This data is representative of three independent experiments (n = 3).

Transwell chamber migration assay was employed to investigate the possible role of MEG3 knockdown (down regulation) in migration of ovarian cancer cell lines after DNC, OXA and combination treatment. Matrigel as membrane model of barrier was used to determine the cell migration capacity by counting the number of cells which able to pass through a porous membrane down to the lower chamber and the degree of their permeability in matrigel in all three treatments after MEG3 knockdown. The density of the cell passed through the matrigel to the other side of the chamber is shown in (Fig. 4). Transwell assay results in previous study had exhibited that all OXA, DNC and combination treatments had a significant inhibitory effect on cell migration in ovarian cancer cell lines compared to untreated control cells. While, MEG3 knockdown notably increased the cell migration from 40 % to 78 % in DNC treatment (p < 0.001), 45 %–60 % in OXA treatment and 25 %–50 % in combination treatment (p <0.001 %). According to these results, it is suggested that the inhibitory effects of DNC and combination treatment on migration capacity of OVCAR3 cells was influenced by MEG3 downregulation. Moreover, the effect of MEG3 downregulation on cell migration in combination treatment was greater than DNC or OXA treatment alone (Fig. 4).

### 3.5 MEG3 down-regulation arrested anti-invasion effect of DNC and oxaliplatin treatment in ovarian cancer cell

**Fig. 5:**
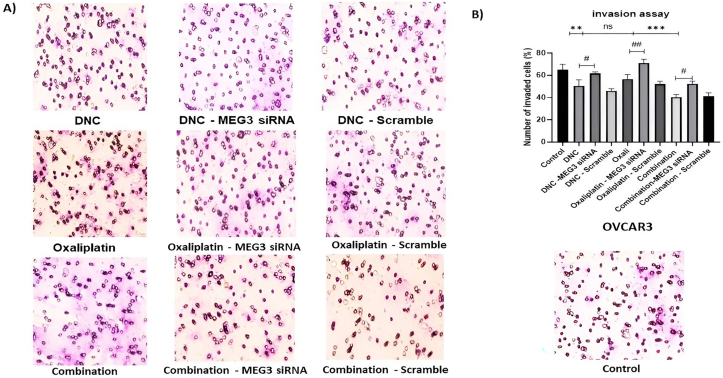
**Effect of MEG3 siRNA on the invasion of OVCAR3 ovarian cancer cells.** (A) Representative images of OVCAR3 cells that have invaded through the matrigel-coated membrane in the Transwell assay. Images are shown for each treatment group, with and without MEG3 siRNA knockdown. (B) Quantitative analysis of cell invasion. The bar graph displays the mean percentage of invading cells ± standard deviation. The effects of DNC, OXA, and the combination treatment were evaluated with and without MEG3 knockdown. The data is from three independent experiments (n = 3). Statistical significance was determined by comparing treated groups to the untreated control group: ∗∗P ≤ 0.01 ∗∗∗P ≤ 0.001, Statistical significance was determined by comparing each treatment group to its corresponding treatment + MEG3 siRNA group: #P ≤ 0.05, ##P ≤ 0.01.

As our previous results, we found that cell invasion was decreased after all 3 treatments in ovarian cancer cell lines. These results revealed that MEG3 knock down (down – regulation) also increased cell invasion from 43 % to 58 % (p < 0.05) in DNC treatment, 55 %–65 % (p < 0.01) in OXA treatment and 40 %–53 % (p <0.05 %) in combination therapy. This finding revealed the significantly effect of MEG3 gene on sensitivity of OVCAR3 cell lines to invasion after all treatments (Fig. 5).

### 3.6 Antagonistic effects of MEG3 knockdown and DNC on cell cycle analysis in OVCAR3 cancer cells

**Fig. 6:**
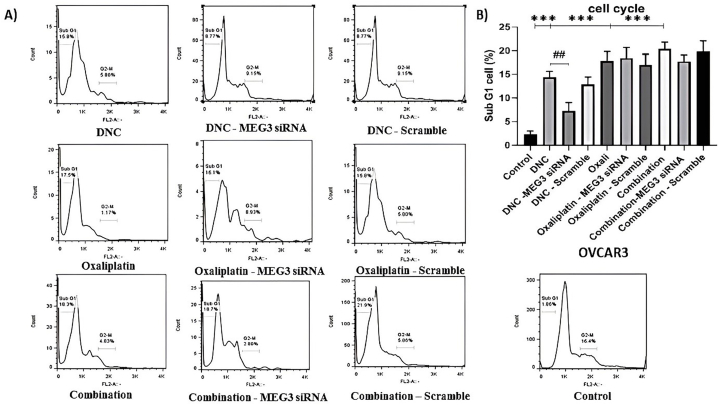
Effect of MEG3 siRNA on DNC, Oxaliplatin, and Combination Therapy in OVCAR3 Ovarian Cancer Cells. (A) Representative flow cytometry histograms showing cell cycle distribution in OVCAR3 cells after treatment with DNC, Oxaliplatin, or their combination, with or without silencing of MEG3 using siRNA. Cells were stained with propidium iodide (PI) and analyzed for DNA content. The percentage of cells in the sub-G1 (apoptotic), G1, and G2-M phases of the cell cycle are indicated. Scramble siRNA was used as a negative control. (B) Quantification of the percentage of cells in the sub-G1 phase for each treatment condition. The data are expressed as the mean ± standard deviation from three independent experiments (n = 3). ∗∗∗P < 0.001 compared to the control group. ##P ≤ 0.01 for comparisons between treatment groups and their corresponding groups with MEG3 siRNA.

In terms of cell cycle analysis, similar to our previous results, all 3 treatments had a significant effect on the cell cycle regulation of OVCAR3 cells (P < 0.001). Notably, our present study found that knockdown of MEG3 remarkably decreased the effect of DNC treatment after 48 h on promoting the G1 phase (P < 0.001) (Fig. 6).

### 3.7 The effect of MEG3 knockdown on the expression of apoptosis and metastasis-associated genes after DNC, OXA treatment and their combination treatment

**Fig. 7:**
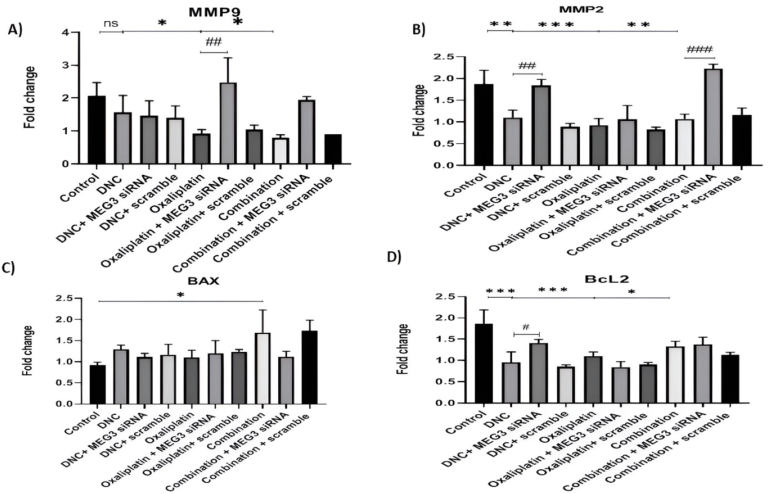
**Effect of MEG3 siRNA on the mRNA expression of MMP9, MMP2, BAX, and BCL2 in OVCAR3 ovarian cancer cells. (A**–**D)** The bar graphs show the fold change in mRNA expression of **(A)** MMP9, (B) MMP2, **(C)** BAX, and **(D)** BCL2 in OVCAR3 cells after treatment with DNC, Oxaliplatin, or their combination. For each treatment, groups with and without MEG3 siRNA, and a scramble siRNA control, were included. Data are presented as the mean ± standard deviation from three independent experiments (n = 3). MMP9: ∗P < 0.05 vs. Control. ##P ≤ 0.01 vs. Oxaliplatin group. MMP2: ∗∗P < 0.01 vs. Control. ##P ≤ 0.01 vs. DNC group. ###P ≤ 0.001 vs. Combination group. BAX: ∗P < 0.05 vs. Combination group. BCL2: ∗∗∗P < 0.001 vs. Control. ∗P < 0.05 vs. DNC group. #P ≤ 0.05 vs. DNC group.

**Fig. 8:**
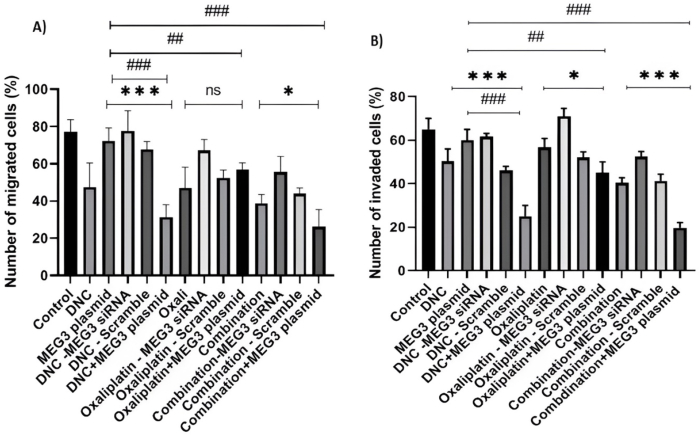
Effect of MEG3 on the migration and invasion of OVCAR3 ovarian cancer cells. (A) This bar graph shows the results of a transwell migration assay, quantifying the percentage of OVCAR3 cells that migrated through a porous membrane. The cells were treated with DNC, Oxaliplatin, or their combination, with or without modulation of MEG3 expression. (B) This bar graph displays the results of a transwell invasion assay, measuring the percentage of cells that invaded through a Matrigel-coated membrane. All data are presented as the mean ± standard deviation from three independent experiments (n = 3). The error bars represent the standard deviation of these repeats. Notes: Data expressed as mean ± standard deviation; ∗P ≤ 0.05, ∗∗P ≤ 0.01, ∗∗∗P ≤ 0.001 treats compared to treats + MEG3 plasmid #P ≤ 0.05, ##P ≤ 0.01, ###P ≤ 0.001 MEG3 plasmid compare to treats + MEG3 plasmid.

We evaluated the function of MEG3 knockdown on associated – apoptosis genes of Bcl2 and BAX as well as matrix metalloproteinase (MMPs) involved in cell metastasis such as MMP-2 and MMP-9. As our previous study, our results revealed reduced expression of MMP2 in OVCAR3 and SKOV3 ovarian cancer cell lines treated with DNC (P < 0.01), OXA (P < 0.001) and combination of them (P < 0.01). While after MEG3 knockdown, MMP2 expression was significantly increased only with DNC (P < 0.01) and combination treatment (P < 0.001). This finding indicated that DNC and combination treatments effect on expression of MMP2 in ovarian cell lines influenced by MEG3 expression. However, MMP9 expression was significantly decreased only after OXA (P < 0.05) and combination treatment (P < 0.05), but not with DNC treatment. After MEG3 knockdown, we found the same results and observed a significant difference in MMP9 expression after OXA treatment, alone (P < 0.01). This result suggested the possible role of MEG3 gene in the treatment of ovarian cancer with OXA which inhibit MMP9-dependent metastasis. In addition, we found no significant difference in the expression of apoptosis-related proteins Bcl-2 and BAX after MEG3 knockdown, suggesting MEG3 didn't influence the apoptosis that was induced by OXA or combination of them in OVCAR3 cells and only Bcl-2 expression increased after treatments and MEG3 knockdown (p < 0.05) (Fig. 7). Considering all these results, we used transwellwss chamber migration and invasion assay to more investigation of the effect of vector–mediated MEG3 over expression. The results further confirmed the remarkable role of the MEG3 over expression in sensitivity of OVCAR3 cells to all 3 treatments (Fig. 8).

## 4 Discussion

During the last decade, the concept of lncRNAs as master regulators of oncogenic signaling has significantly reshaped our understanding of tumor biology (Mishra et al., 2019; Ordónez-Rubiano et al., 2024). It is now well established that lncRNAs contribute to a wide spectrum of cancer hallmarks including sustained proliferation, evasion of apoptosis, invasion, and drug resistance (Saleh et al., 2024; Tufail et al., 2024; Liu et al., 2021). Among these transcripts, MEG3 has emerged as one of the most consistent tumor suppressors, with numerous studies associating its downregulation with poor prognosis and aggressive behavior in different malignancies, including ovarian carcinoma (Seyed Hosseini et al., 2019). Evidence from clinical samples of epithelial ovarian cancer has shown that low MEG3 expression correlates with advanced stage disease, high grade pathology, and unfavorable survival outcomes, while higher levels of MEG3 are associated with improved therapeutic response and better prognosis (Xu et al., 2021). These clinical findings align closely with our experimental results, which demonstrate that treatment with DNC, OXA and their combination significantly increased MEG3 expression in OVCAR3 cells. The elevation of MEG3 was particularly pronounced at 48 h relative to 24 h, and OXA alone elicited a stronger induction than the combination treatment (Seyed Hosseini et al., 2023). This suggests that MEG3 upregulation may be a downstream transcriptional or epigenetic response to chemotherapeutic stress, potentially involving activation of tumor suppressive signaling pathways such as p53 or PTEN (Xu et al., 2022; Assal et al., 2025).

The interplay between MEG3 expression and apoptotic regulation provides an important mechanistic explanation for these results (Zhang et al., 2019). Our prior work revealed that DNC alone modulated the expression of pro- and anti-apoptotic markers BAX and Bcl-2 in MEG3 knockdown cells, suggesting that MEG3 may specifically facilitate curcumin-derived apoptotic signaling (Seyed Hosseini et al., 2019). Comparable findings have been reported in other contexts; for example, Shi and colleagues demonstrated that curcumin induced an increase in BAX and alterations in Bcl-2 family proteins in ovarian cancer, supporting the notion that phytochemical agents can enhance apoptosis via MEG3-associated axes (Shi et al., 2006). Interestingly, in our present experiments, MEG3 knockdown did not significantly change Bax expression under any of the treatment conditions, suggesting that MEG3's contribution to apoptosis may be mediated through different downstream factors such as p53 transcriptional targets, or via modulation of mitochondrial stability through Bcl-XL rather than Bax. Indeed, earlier investigations in lung adenocarcinoma showed that MEG3 overexpression enhanced cisplatin sensitivity through p53-dependent regulation and downregulation of Bcl-XL, supporting the idea that MEG3's apoptotic regulation may vary across tumor contexts but is consistently associated with improved drug responsiveness (Zhang et al., 2023). The role of MEG3 in regulating invasion and metastasis is equally compelling. Our results demonstrated that silencing MEG3 significantly increased MMP-2 expression after DNC and combination treatment, while OXA alone induced a rise in MMP-9 expression. Since matrix metalloproteinases are key enzymes in extracellular matrix degradation and metastatic dissemination, these changes strongly suggest that MEG3 normally suppresses invasive potential, and that its downregulation facilitates cell migration (Eble and Niland, 2019). Elevated MMP-2 and MMP-9 have been consistently correlated with poor outcomes and metastatic progression in ovarian and other cancers, and our observations reinforce this established paradigm (Jiang and Li, 2021). Mechanistically, MEG3 may exert this suppressive role through epigenetic silencing of pro-metastatic genes or through competing endogenous RNA networks that regulate metastasis-associated microRNAs (Liu et al., 2021). For instance, studies have shown that MEG3 can regulate angiogenesis and invasion through modulation of miRNA targets such as miR-885-5p, leading to upregulation of angiogenesis inhibitors (Zheng and Hou, 2021). In ovarian cancer, MEG3 overexpression has been repeatedly shown to decrease migration and invasion, further supporting our finding that MEG3 deficiency permits higher expression of MMPs and greater invasive activity (Sun et al., 2016). Analysis of the cell cycle adds another dimension to MEG3's regulatory functions. In MEG3-proficient cells, treatments with DNC, OXA, or their combination increased sub-G1 phase accumulation, a marker of DNA fragmentation and apoptosis. However, MEG3 knockdown reduced this accumulation, particularly suppressing the sub-G1 increase induced by DNC (Awasthee et al., 2023). This indicates that MEG3 contributes to drug-induced apoptotic cell cycle arrest, potentially through regulation of cyclin-dependent kinase inhibitors or checkpoint proteins such as CDC2 and CDC25A (Chatterjee and Viswanathan, 2021; Liu et al., 2020). Previous reports have highlighted MEG3's ability to modulate p53 and RB1 pathways, further supporting the possibility that MEG3 orchestrates transcriptional programs controlling the cell cycle (He et al., 2017b). The diminished clonogenic suppression and altered migration patterns observed after MEG3 silencing in our study also corroborate its critical role in limiting proliferative and invasive potential under chemotherapeutic stress (Kruer et al., 2016). When the different strands of evidence are integrated, a coherent model emerges in which MEG3 acts as a central mediator linking chemotherapy agents to multiple facets of ovarian cancer cell biology. Its upregulation by DNC and OXA contributes to apoptosis through modulation of Bcl-2 family proteins and possibly p53-related targets, restricts invasion by suppressing MMP-2 and MMP-9, and enforces cell cycle arrest to limit proliferation (D Amaral et al., 2010). Knockdown of MEG3 abrogates these beneficial effects, reduces sensitivity to chemotherapy, and promotes a more aggressive and invasive phenotype. Importantly, MEG3 silencing also abolished the synergistic benefit of combined DNC and OXA, underscoring its indispensable role in mediating enhanced drug responsiveness (Assal et al., 2025; He et al., 2017b). These findings have important implications for translational oncology. First, they support the notion that MEG3 can be harnessed as a biomarker for both prognosis and prediction of chemotherapy response in ovarian cancer patients. Measuring MEG3 levels before and during treatment could help identify patients more likely to benefit from specific chemotherapeutic regimens. Second, therapeutic strategies aimed at restoring MEG3 function may augment drug sensitivity. Delivery of MEG3 transcripts, modulation of epigenetic regulators that control its expression, or targeting the upstream regulators such as METTL3/YTHDF2 that mediate its stability are promising approaches. Third, further mechanistic studies are needed to delineate the precise apoptotic and cell cycle signaling pathways under MEG3 control in ovarian cancer, as well as to map the microRNA and epigenetic networks that mediate its influence on metastasis. Finally, validation of our findings in animal xenograft models would provide a critical step toward clinical translation, offering insights into whether MEG3 restoration could potentiate the effects of existing chemotherapeutics in vivo. Altogether, the data presented here reinforce MEG3 as a multifunctional tumor suppressor that orchestrates apoptosis, cell cycle arrest, invasion, and therapeutic response in ovarian cancer cells treated with DNC and OXA. By revealing how MEG3 knockdown diminishes apoptotic sensitivity, enhances invasive capacity through MMP regulation, and abolishes drug synergy, our work positions MEG3 as a promising target for therapeutic exploitation. Integrating MEG3 modulation into ovarian cancer treatment strategies holds potential not only for improving the efficacy of conventional drugs but also for curbing metastasis and prolonging patient survival.

## 5 Conclusion

The present investigation highlights MEG3 as a central determinant of ovarian cancer cell responses to DNC, OXA and their combination, revealing that the tumor-suppressive lncRNA actively governs gene expression programs linked to apoptosis, invasion, and cell-cycle regulation. By positioning MEG3 as a mediator of drug sensitivity, our study not only consolidates its role in tumor biology but also suggests that the efficacy of both nanotechnology-based and conventional therapies is tightly interwoven with the lncRNA regulatory landscape. These findings point toward the therapeutic potential of combining epigenetic or lncRNA-targeted strategies with existing chemotherapeutic agents to overcome one of the most pressing clinical challenges in ovarian cancer management—chemoresistance. Importantly, the results also underscore the concept that modulation of a single lncRNA such as MEG3 can reverberate through multiple oncogenic pathways, altering the equilibrium between apoptosis and survival, metastasis and containment, or chemosensitivity and resistance. This highlights MEG3 not merely as a biomarker of drug responsiveness but also as a strategic therapeutic node, offering opportunities to fine-tune treatment regimens. Incorporating MEG3 modulation could enhance the cytotoxic potential of platinum compounds while mitigating metastatic progression through MMP regulation, potentially leading to more durable therapeutic responses. Nonetheless, the clinical translation of these findings necessitates careful validation. Our study relied on in vitro systems, which, although highly informative, cannot fully capture the complexity of tumor heterogeneity, stromal interactions, or immune modulation inherent in the ovarian cancer microenvironment. Therefore, future efforts must extend into in vivo models to delineate how MEG3 expression dynamics influence tumor growth, metastatic dissemination, and treatment responsiveness in a physiological setting. Such studies should also explore pharmacological or nanocarrier-based methods for restoring or stabilizing MEG3 levels, ensuring practical applicability in clinical oncology. Moreover, this work raises the broader possibility that DNC and OXA, beyond their established cytotoxic properties, may exert therapeutic benefits in part by reshaping the non-coding transcriptome. Systematic mapping of lncRNA networks under these treatment conditions could reveal a hierarchy of non-coding RNAs that collectively govern ovarian cancer behavior, with MEG3 occupying a central but not solitary position. Integration of transcriptomic profiling, epigenetic analyses, and functional assays will be critical to identifying additional lncRNAs that may act synergistically with MEG3 or compensate for its loss. Overall, this investigation identifies MEG3 as a critical regulatory molecule as well as a promising therapeutic target in ovarian cancer management. By linking nanocurcumin- and oxaliplatin-induced cytotoxicity to lncRNA regulation, it establishes a novel conceptual framework in which non-coding RNA biology intersects with nanotechnology and chemotherapy. This integrative approach holds promise for developing more effective, less resistant, and personalized therapeutic strategies for ovarian cancer patients.

## Consent to publish

Not applicable.

## Ethics approval and consent to participate

Not applicable.

## Credit author statement

Elahe Seyed Hosseini: Conceptualization, Methodology, Investigation, Writing – Original Draft.

Marziyeh Alizadeh Zarei: Methodology, Investigation, Writing – Original Draft.

Hossein Nikzad: Supervision, Methodology, Guidance, Writing – Original Draft.

Hamed Haddad Kashani: Writing – Original Draft, Literature Review, Writing – Review & Editing.

Zahra Shabani: Writing – Review & Editing, Critical Revision.

All authors have read and approved the final manuscript.

## Funding

The present work was financially supported by grant no. No. 95160 from Kashan University of Medical Sciences, Kashan, Iran.

## Declaration of competing interest

The authors declare that they have no known competing financial interests or personal relationships that could have appeared to influence the work reported in this paper.

## Data Availability

All the authors confirm the availability of data and materials.

## References

[bib1] Assal R.A., Rashwan H.H., Zakaria Z.I., Sweillam J.H., Fouda Y.M., Abdelhamid A.M., Youness R.A. (2025). Deciphering the mysteries of MEG3 LncRNA and its implications in genitourinary cancers. Front. Oncol..

[bib2] Awasthee N., Shekher A., Rai V., Verma S.S., Mishra S., Gupta S.C. (2023). https://scholarworks.utrgv.edu/somrs/theme1/track1/28.

[bib3] Babaei E., Sadeghizadeh M., Hassan Z.M., Feizi M.A.H., Najafi F., Hashemi S.M. (2012). Dendrosomal curcumin significantly suppresses cancer cell proliferation in vitro and in vivo. Int. Immunopharmacol..

[bib4] Chatterjee M., Viswanathan P. (2021). Long noncoding RNAs in the regulation of p53‐mediated apoptosis in human cancers. Cell Biol. Int..

[bib5] Choudhuri T., Pal S., Das T., Sa G. (2005). Curcumin selectively induces apoptosis in deregulated cyclin D1-expressed cells at G2 phase of cell cycle in a p53-dependent manner. J. Biol. Chem..

[bib6] D Amaral J., M Xavier J., J Steer C., Mp Rodrigues C. (2010). Targeting the p53 pathway of apoptosis. Curr. Pharm. Des..

[bib7] Eble J.A., Niland S. (2019). The extracellular matrix in tumor progression and metastasis. Clin. Exp. Metastasis.

[bib8] Fanelli M.F., Chinen L.T.D., Begnami M.D., Costa Jr WL., Fregnami J.H.T., Soares F.A., Montagnini A.L. (2012). The influence of transforming growth factor‐α, cyclooxygenase‐2, matrix metalloproteinase (MMP)‐7, MMP‐9 and CXCR4 proteins involved in epithelial–mesenchymal transition on overall survival of patients with gastric cancer. Histopathology.

[bib9] Ferlay J., Shin H.R., Bray F., Forman D., Mathers C., Parkin D.M. (2010). Estimates of worldwide burden of cancer in 2008: globocan 2008. Int. J. Cancer.

[bib10] Ghafouri-Fard S., Taheri M. (2019). Maternally expressed gene 3 (MEG3): a tumor suppressor long non coding RNA. Biomed. Pharmacother..

[bib11] Gou M., Men K., Shi H., Xiang M., Zhang J., Song J., Long J., Wan Y., Luo F., Zhao X. (2011). Curcumin-loaded biodegradable polymeric micelles for Colon cancer therapy in vitro and in vivo. Nanoscale.

[bib12] Gupta S.C., Patchva S., Aggarwal B.B. (2013). Therapeutic roles of curcumin: lessons learned from clinical trials. AAPS J..

[bib13] Gupta M.K., Sansare V., Shrivastava B., Jadhav S., Gurav P. (2022). Fabrication and evaluation of mannose decorated curcumin loaded nanostructured lipid carriers for hepatocyte targeting: in vivo hepatoprotective activity in Wistar rats. Curr. Res. Pharmacol. Drug Discov..

[bib14] He B., Wei W., Liu J., Xu Y., Zhao G. (2017). Synergistic anticancer effect of curcumin and chemotherapy regimen FP in human gastric cancer MGC-803 cells. Oncol. Lett..

[bib15] He Y., Luo Y., Liang B., Ye L., Lu G., He W. (2017). Potential applications of MEG3 in cancer diagnosis and prognosis. Oncotarget.

[bib16] Hosseini E.S., Meryet-Figuiere M., Sabzalipoor H., Kashani H.H., Nikzad H., Asemi Z. (2017). Dysregulated expression of long noncoding RNAs in gynecologic cancers. Mol. Cancer.

[bib17] Hosseini E.S., Zarei M.A., Babashah S., Sistani R.N., Sadeghizadeh M., Kashani H.H., Mahabadi J.A., Izadpanah F., Atlasi M.A., Nikzad H. (2019). Studies on combination of oxaliplatin and dendrosomal nanocurcumin on proliferation, apoptosis induction, and long non-coding RNA expression in ovarian cancer cells. Cell Biol. Toxicol..

[bib18] Hosseini E.S., Zarei M.A., Kashani H.H., Salimian M., Kashani N.R., Nikzad H. (2021). Altered long non-coding RNAs expression and cytotoxic and anti-proliferative activity of Dendrosomal nano-curcumin in ovarian cancer cells. Indian J.Gynecologic Oncology.

[bib19] Jiang H., Li H. (2021). Prognostic values of tumoral MMP2 and MMP9 overexpression in breast cancer: a systematic review and meta-analysis. BMC Cancer.

[bib20] Kruer T.L., Dougherty S.M., Reynolds L., Long E., de Silva T., Lockwood W.W., Clem B.F. (2016). Expression of the lncRNA maternally expressed gene 3 (MEG3) contributes to the control of lung cancer cell proliferation by the Rb pathway. PLoS One.

[bib21] Kunnumakkara A.B., Anand P., Aggarwal B.B. (2008). Curcumin inhibits proliferation, invasion, angiogenesis and metastasis of different cancers through interaction with multiple cell signaling proteins. Cancer Lett..

[bib22] Li W., Li S., Deng L., Yang S., Li M., Long S., Chen S., Lin F., Xiao L. (2015). Decreased MT1-MMP in gastric cancer suppressed cell migration and invasion via regulating MMPs and EMT. Tumor Biol..

[bib23] Liu K., Zheng M., Lu R., Du J., Zhao Q., Li Z., Li Y., Zhang S. (2020). The role of CDC25C in cell cycle regulation and clinical cancer therapy: a systematic review. Cancer Cell Int..

[bib24] Liu S.J., Dang H.X., Lim D.A., Feng F.Y., Maher C.A. (2021). Long noncoding RNAs in cancer metastasis. Nat. Rev. Cancer.

[bib25] Machover D., Diaz-Rubio E., De Gramont A., Schilf A., Gastiaburu J.-J., Brienza S., Itzhaki M., Metzger G., N'daw D., Vignoud J. (1996). Two consecutive phase II studies of oxaliplatin (L-OHP) for treatment of patients with advanced colorectal carcinoma who were resistant to previous treatment with fluoropyrimidines. Ann. Oncol..

[bib26] Miller K.D., Siegel R.L., Lin C.C., Mariotto A.B., Kramer J.L., Rowland J.H., Stein K.D., Alteri R., Jemal A. (2016). Cancer treatment and survivorship statistics, 2016. CA Cancer J. Clin..

[bib27] Mishra S., Verma S.S., Rai V., Awasthee N., Chava S., Hui K.M., Kumar A.P., Challagundla K.B., Sethi G., Gupta S.C. (2019). Long non-coding RNAs are emerging targets of phytochemicals for cancer and other chronic diseases. Cell. Mol. Life Sci..

[bib28] Ordónez-Rubiano E.G., Rincón-Arias N., Espinosa S., Shelton W.J., Salazar A.F., Cómbita A., Baldoncini M., Luzzi S., Payán-Gómez C., Gómez-Amarillo D.F. (2024). The potential of miRNA-based approaches in glioblastoma: an update in current advances and future perspectives. Curr. Res. Pharmacol. Drug Discov..

[bib29] Qin R., Chen Z., Ding Y., Hao J., Hu J., Guo F. (2013). Long non-coding RNA MEG3 inhibits the proliferation of cervical carcinoma cells through the induction of cell cycle arrest and apoptosis. Neoplasma.

[bib30] Radisky E.S., Radisky D.C. (2010). Matrix metalloproteinase-induced epithelial-mesenchymal transition in breast cancer. J. Mammary Gland Biol. Neoplasia.

[bib31] Sadeghizadeh M., Ranjbar B., Damaghi M., Khaki L., Sarbolouki M.N., Najafi F., Parsaee S., Ziaee A.A., Massumi M., Lubitz W. (2008). Dendrosomes as novel gene porters‐III. J. Chem. Technol. Biotechnol. Int. Res. Process Environ. Clean Technol..

[bib32] Saleh R.O., Al-Ouqaili M.T., Ali E., Alhajlah S., Kareem A.H., Shakir M.N., Alasheqi M.Q., Mustafa Y.F., Alawadi A., Alsaalamy A. (2024). lncRNA-microRNA axis in cancer drug resistance: particular focus on signaling pathways. Med. Oncol..

[bib33] Sarbolouki M.N., Sadeghizadeh M., Yaghoobi M.M., Karami A., Lohrasbi T. (2000). Dendrosomes: a novel family of vehicles for transfection and therapy. J. Chem. Technol. Biotechnol. Int. Res. Process Environ. Clean Technol..

[bib34] Sato H., Takino T., Okada Y., Cao J., Shinagawa A., Yamamoto E., Seiki M. (1994). A matrix metalloproteinase expressed on the surface of invasive tumour cells. Nature.

[bib35] Seyed Hosseini E., Alizadeh Zarei M., Babashah S., Nakhaei Sistani R., Sadeghizadeh M., Haddad Kashani H., Amini Mahabadi J., Izadpanah F., Atlasi M.A., Nikzad H. (2019). Studies on combination of oxaliplatin and dendrosomal nanocurcumin on proliferation, apoptosis induction, and long non-coding RNA expression in ovarian cancer cells. Cell Biol. Toxicol..

[bib36] Seyed Hosseini E., Alizadeh Zarei M., Tarrahimofrad H., Zamani J., Haddad Kashani H., Ahmad E., Nikzad H. (2023). Synergistic effects of dendrosomal nanocurcumin and oxaliplatin on oncogenic properties of ovarian cancer cell lines by down-expression of MMPs. Biol. Res..

[bib37] Shi M., Cai Q., Yao L., Mao Y., Ming Y., Ouyang G. (2006). Antiproliferation and apoptosis induced by curcumin in human ovarian cancer cells. Cell Biol. Int..

[bib38] Shishodia S., Chaturvedi M.M., Aggarwal B.B. (2007). Role of curcumin in cancer therapy. Curr. Probl. Cancer.

[bib39] Spizzo R., Almeida MIe, Colombatti A., Calin G.A. (2012). Long non-coding RNAs and cancer: a new frontier of translational research?. Oncogene.

[bib40] Sun L., Li Y., Yang B. (2016). Downregulated long non-coding RNA MEG3 in breast cancer regulates proliferation, migration and invasion by depending on p53's transcriptional activity. Biochem. Biophys. Res. Commun..

[bib41] Tufail M., Hu J.-J., Liang J., He C.-Y., Wan W.-D., Huang Y.-Q., Jiang C.-H., Wu H., Li N. (2024). Hallmarks of cancer resistance. iScience.

[bib42] Wang P., Ren Z., Sun P. (2012). Overexpression of the long non‐coding RNA MEG3 impairs in vitro glioma cell proliferation. J. Cell. Biochem..

[bib43] Xu X., Zhong Z., Shao Y., Yi Y. (2021). Prognostic value of MEG3 and its correlation with immune infiltrates in gliomas. Front. Genet..

[bib44] Xu J., Wang X., Zhu C., Wang K. (2022). A review of current evidence about lncRNA MEG3: a tumor suppressor in multiple cancers. Front. Cell Dev. Biol..

[bib45] Ying L., Huang Y., Chen H., Wang Y., Xia L., Chen Y., Liu Y., Qiu F. (2013). Downregulated MEG3 activates autophagy and increases cell proliferation in bladder cancer. Mol. Biosyst..

[bib46] Zamani M., Sadeghizadeh M., Behmanesh M. (2014). Dendrosomal curcumin upregulates expression of the long non-coding RNA gene MEG3 in U87MG glioblastoma cells. Pathobiology Research.

[bib47] Zamani M., Sadeghizadeh M., Behmanesh M., Najafi F. (2015). Dendrosomal curcumin increases expression of the long non-coding RNA gene MEG3 via up-regulation of epi-miRs in hepatocellular cancer. Phytomedicine.

[bib48] Zarei M.A., Nikzad H., Shabani Z., Kashani H.H., Hosseini E.S. (2025). Dendrosomal curcumin nanoformulation induces apoptosis via Bax/Bcl-2 in human ovarian cancer OVCAR3 cells. Cancer Treat. Res. Commun..

[bib49] Zhang X., Rice K., Wang Y., Chen W., Zhong Y., Nakayama Y., Zhou Y., Klibanski A. (2010). Maternally expressed gene 3 (MEG3) noncoding ribonucleic acid: isoform structure, expression, and functions. Endocrinology.

[bib50] Zhang Y., Wu J., Jing H., Huang G., Sun Z., Xu S. (2019). Long noncoding RNA MEG3 inhibits breast cancer growth via upregulating endoplasmic reticulum stress and activating NF‐κB and p53. J. Cell. Biochem..

[bib51] Zhang Z., Shi S., Li J., Costa M. (2023). Long non-coding RNA MEG3 in metal carcinogenesis. Toxics.

[bib52] Zheng Q., Hou W. (2021). Regulation of angiogenesis by microRNAs in cancer. Mol. Med. Rep..

